# Supplementary material for: A Likelihood Approach for Real-Time Calibration of Stochastic Compartmental Epidemic Models
Source: PLoS Comput Biol. 2017 Jan 17;13(1):e1005257. doi: 10.1371/journal.pcbi.1005257 (PMC5240920; doi:10.1371/journal.pcbi.1005257)
Supplement: S1 File — (TAR.GZ) [file pcbi.1005257.s014.tar.gz › HSPH_Online-SI-Revision/EnKF07/n1-extreme/n1-extreme_EnKF_table8.pdf]

1	R0	{2.95019, 14.2854}	{2.39773, 5.13574}	{{0.908723, 1.44169}, {3., 8.76208}}	{20.9212, 64.049}	inf	19.	95.7144	{5.40008, 275.707}	433.874	0.448545	newly	10.	104.19	{5., 437.}	961.3	0.62527	Reff	0.253716	0.460319	{0.0324879, 0.96676}	126.973	ar	924.	1786.22	{1530., 2696.}	93.3139
2	R0	{2.67565, 16.946}	{2.85423, 4.94638}	{{2.44426, 2.44098}, {3., 10.2256}}	{12.081, 71.144}	inf	31.	31.7057	{2.95043, 42.6064}	100.469	0.495819	newly	14.	62.44	{3., 106.}	386.857	0.437773	Reff	0.232782	0.604718	{0.190799, 1.6694}	169.047	ar	926.	1635.06	{1484., 2035.}	76.5724
3	R0	{2.40066, 16.6543}	{2.55235, 6.68717}	{{2.02837, 3.26849}, {3., 10.0164}}	{24.0116, 59.8472}	inf	69.	63.2048	{9.99153, 57.5401}	91.4971	0.475794	newly	26.	58.1	{6., 69.}	171.846	0.380264	Reff	0.249669	0.455562	{0.171962, 0.913875}	100.19	ar	916.	1692.1	{1535., 1894.}	84.7271
4	R0	{1.9294, 15.4361}	{2.39861, 5.9162}	{{1.31246, 3.37379}, {3., 10.719}}	{45.6216, 61.673}	inf	64.	88.4836	{12.1914, 148.847}	125.132	0.443071	newly	26.	91.29	{8., 206.}	284.654	0.409309	Reff	0.507432	0.540632	{0.160188, 1.10344}	53.2291	ar	796.	1760.08	{1527., 2362.}	121.116
5	R0	{2.92859, 19.5393}	{2.6613, 8.23311}	{{1.33093, 2.62283}, {3., 19.2098}}	{12.9309, 57.8708}	inf	46.	51.45	{8.25634, 71.8393}	87.9878	0.373492	newly	14.	38.07	{6., 58.}	200.357	0.410744	Reff	0.251859	0.473893	{0.111386, 0.953862}	117.317	ar	927.	1558.42	{1394., 1949.}	68.1143
6	R0	{1.79622, 12.3148}	{2.65456, 7.69178}	{{0.888385, 4.92896}, {3., 12.3421}}	{61.069, 37.7621}	inf	28.	106.045	{6.15937, 185.703}	306.836	0.483047	newly	16.	74.94	{5., 189.}	394.5	0.555401	Reff	0.555032	0.488962	{0.125814, 0.903135}	61.003	ar	734.	1788.68	{1542., 2298.}	143.689
7	R0	{2.45855, 17.1776}	{2.72225, 5.3803}	{{2.43971, 0.330304}, {3., 16.4715}}	{13.4998, 68.6784}	inf	47.	86.8688	{13.1112, 117.698}	150.862	0.372467	newly	18.	110.18	{13., 260.}	528.222	0.464906	Reff	0.358948	0.651481	{0.305506, 1.43683}	93.5996	ar	871.	1831.48	{1586., 2514.}	110.273
8	R0	{2.20009, 15.519}	{2.32637, 4.81117}	{{0.792631, 1.22988}, {3., 5.83639}}	{42.0356, 68.9982}	inf	24.	46.4677	{8.31392, 85.0807}	134.858	0.282084	newly	12.	46.34	{6., 143.}	306.333	0.397755	Reff	0.378415	0.368096	{0.0774524, 0.790009}	47.2246	ar	839.	1641.64	{1490., 2030.}	95.6663
9	R0	{2.06493, 9.6414}	{2.39193, 6.05794}	{{1.02404, 1.96125}, {3., 12.452}}	{45.0221, 49.7785}	inf	11.	48.927	{6.12086, 65.5251}	363.103	0.541523	newly	7.	52.11	{5., 100.}	656.714	0.632818	Reff	0.489388	0.463969	{0.134063, 0.743692}	53.26	ar	777.	1678.79	{1533., 2008.}	116.06
10	R0	{2.60775, 19.6936}	{2.37449, 10.0503}	{{0.578322, 4.23526}, {3., 15.3799}}	{31.1963, 48.968}	inf	57.	90.1851	{15.5114, 93.2322}	106.346	0.294425	newly	14.	67.98	{9., 190.}	402.429	0.47643	Reff	0.341615	0.558741	{0.123658, 0.774615}	99.6857	ar	889.	1661.69	{1446., 2326.}	86.9168
11	R0	{1.99168, 8.13555}	{2.75045, 6.83047}	{{2.42046, 5.80253}, {3., 8.71633}}	{39.2982, 19.519}	inf	7.	72.9238	{8.09808, 137.186}	947.638	0.707516	newly	6.	77.77	{6., 250.}	1202.83	0.713138	Reff	0.478003	0.655916	{0.236126, 1.65296}	69.7144	ar	772.	1787.77	{1567., 2343.}	131.576
12	R0	{1.79304, 9.18941}	{2.39693, 5.61115}	{{0.942222, 0.33278}, {3., 11.4059}}	{60.416, 50.9347}	inf	15.	52.3883	{7.7793, 53.0025}	269.407	0.402858	newly	9.	106.93	{6., 358.}	1103.22	0.586674	Reff	0.505637	0.539164	{0.0791536, 1.11052}	69.179	ar	725.	1710.07	{1540., 2236.}	135.872
13	R0	{1.84016, 18.3431}	{2.32341, 3.33803}	{{0.535696, 2.0243}, {3., 3.54788}}	{64.501, 81.8022}	inf	80.	113.064	{12.459, 384.495}	117.303	0.442099	newly	33.	154.27	{13., 521.}	389.242	0.504075	Reff	0.651417	0.529328	{0.132427, 0.703979}	33.3574	ar	762.	1758.89	{1526., 2316.}	130.825
14	R0	{1.94221, 9.85971}	{2.59931, 7.31697}	{{1.34815, 3.35314}, {3., 10.4078}}	{48.6333, 30.5765}	inf	4.	68.0663	{7.64984, 71.044}	1604.7	0.993243	newly	2.	57.56	{8., 64.}	2780.	1.16764	Reff	0.388442	0.439993	{0.0896986, 0.711302}	61.0117	ar	805.	1681.52	{1542., 1736.}	108.884
15	R0	{1.78578, 12.0541}	{2.87834, 2.31221}	{{2.39168, 0.565134}, {3., 4.22343}}	{61.1809, 80.8181}	inf	20.	20.0893	{4.44403, 25.6574}	77.7391	0.322124	newly	12.	40.15	{8., 58.}	249.917	0.323934	Reff	0.494661	0.499411	{0.213146, 0.67405}	41.4003	ar	732.	1548.38	{1426., 1772.}	111.527
16	R0	{1.94463, 15.6503}	{2.73367, 7.4306}	{{2.44631, 2.4873}, {3., 18.3642}}	{41.9434, 59.4339}	inf	49.	68.0614	{5.23127, 104.054}	111.212	0.429673	newly	20.	92.72	{4., 257.}	407.	0.5503	Reff	0.536718	0.630154	{0.13799, 1.4698}	64.3287	ar	757.	1726.91	{1513., 2208.}	128.125
17	R0	{2.08901, 16.4314}	{2.38233, 13.0702}	{{0.144568, 8.16252}, {3., 18.0548}}	{52.2167, 26.2946}	inf	74.	39.5063	{8.42101, 73.8342}	57.3302	0.437142	newly	26.	19.04	{3., 35.}	54.9231	0.362517	Reff	0.48465	0.363252	{0.025727, 0.526127}	48.5116	ar	826.	1548.29	{1471., 1620.}	87.4443
18	R0	{2.76419, 18.56}	{2.7245, 6.43886}	{{2.38549, 3.65752}, {3., 9.0728}}	{11.4499, 65.3398}	inf	52.	53.8043	{10.5946, 80.4195}	77.5916	0.37252	newly	13.	68.05	{8., 128.}	442.538	0.512886	Reff	0.243249	0.647394	{0.249665, 1.60012}	178.622	ar	920.	1725.29	{1548., 1996.}	87.5315
19	R0	{2.3821, 17.1399}	{2.43961, 7.29767}	{{1.10865, 1.13114}, {3., 12.1855}}	{31.9637, 57.4229}	inf	44.	66.4145	{11.9918, 149.995}	103.338	0.301209	newly	11.	84.93	{6., 267.}	688.091	0.547095	Reff	0.32873	0.433655	{0.0703779, 0.724789}	74.7256	ar	883.	1750.43	{1599., 2079.}	98.2367
20	R0	{2.78314, 19.6127}	{2.27248, 7.81281}	{{0.597159, 2.03703}, {3., 15.9368}}	{28.9455, 60.1645}	inf	20.	129.858	{15.3008, 528.208}	560.175	0.54824	newly	5.	122.32	{10., 510.}	2351.2	0.962236	Reff	0.18647	0.400281	{0.0910826, 0.513572}	140.81	ar	943.	1770.53	{1518., 2749.}	87.755
21	R0	{1.85124, 14.6794}	{2.40986, 7.56076}	{{0.372151, 3.35259}, {3., 10.6626}}	{65.5882, 51.9636}	inf	67.	76.2296	{13.4122, 76.6717}	89.9759	0.344031	newly	31.	65.53	{10., 97.}	155.645	0.318422	Reff	0.596099	0.453339	{0.0477179, 0.688545}	56.4408	ar	730.	1708.9	{1543., 1994.}	134.096
22	R0	{2.1028, 14.9852}	{2.60703, 9.22702}	{{1.52666, 3.33389}, {3., 13.2582}}	{38.7562, 40.7257}	inf	31.	68.3326	{6.4308, 109.866}	158.895	0.398614	newly	11.	47.42	{4., 78.}	356.364	0.543267	Reff	0.395326	0.607898	{0.152157, 1.12276}	75.9811	ar	840.	1731.14	{1539., 2166.}	106.088
23	R0	{1.86601, 10.2157}	{2.73007, 4.99012}	{{2.42319, 1.08362}, {3., 11.4171}}	{50.7456, 56.0881}	inf	19.	44.6624	{8.70018, 79.6064}	162.248	0.38936	newly	9.	94.04	{7., 195.}	956.	0.657997	Reff	0.546741	0.617469	{0.270807, 1.44688}	52.9446	ar	747.	1710.41	{1545., 2056.}	128.971
24	R0	{2.34032, 10.0992}	{2.3763, 5.72837}	{{0.903648, 1.29306}, {3., 12.4757}}	{36.8023, 54.5934}	inf	19.	52.0108	{5.2864, 118.514}	201.918	0.355022	newly	7.	78.06	{5., 156.}	1029.14	0.648243	Reff	0.278498	0.506486	{0.0859301, 0.934796}	119.528	ar	887.	1715.95	{1542., 2254.}	93.4555
25	R0	{1.91548, 15.3401}	{2.3385, 4.45398}	{{1.26673, 1.23754}, {3., 9.06852}}	{47.8449, 70.9651}	inf	36.	52.4127	{2.73876, 102.155}	143.064	0.558221	newly	14.	66.49	{2., 209.}	422.357	0.519091	Reff	0.599545	0.48481	{0.0433383, 0.995753}	53.093	ar	765.	1682.18	{1510., 2181.}	119.893
26	R0	{2.75283, 10.6721}	{2.80241, 7.56073}	{{2.34684, 1.72622}, {3., 14.3802}}	{10.65, 44.0987}	inf	11.	56.2463	{4.90111, 34.8871}	430.406	0.455959	newly	5.	36.8	{4., 40.}	646.8	0.606442	Reff	0.225732	0.506262	{0.275915, 0.688156}	131.487	ar	926.	1688.53	{1531., 1832.}	82.3467
27	R0	{2.08367, 19.7849}	{2.61515, 4.23419}	{{2.15419, 2.94574}, {3., 5.48503}}	{39.2839, 78.5989}	inf	70.	34.3862	{2.77797, 32.9378}	98.6725	0.827901	newly	16.	47.13	{2., 51.}	251.438	0.454072	Reff	0.43132	0.351956	{0.0606483, 0.467987}	53.5925	ar	833.	1612.81	{1492., 1774.}	93.6146
28	R0	{2.64334, 17.1765}	{2.63689, 4.99509}	{{2.01472, 0.445968}, {3., 9.20675}}	{17.9672, 71.1556}	inf	50.	58.6258	{3.62382, 149.105}	122.889	0.620567	newly	17.	67.49	{4., 270.}	343.353	0.509497	Reff	0.227327	0.521955	{0.0702832, 1.25862}	167.444	ar	934.	1718.01	{1503., 2324.}	83.9411
29	R0	{2.87008, 13.3872}	{2.85275, 6.47728}	{{2.28743, 1.41471}, {3., 14.7115}}	{7.57488, 55.7278}	inf	16.	33.5844	{3.95588, 28.9262}	165.665	0.349477	newly	8.	71.87	{1., 50.}	835.375	0.56187	Reff	0.172205	0.673367	{0.0597277, 2.19694}	324.227	ar	941.	1649.63	{1464., 2325.}	75.3061
30	R0	{2.06105, 19.6275}	{2.40409, 7.88702}	{{0.999225, 4.23281}, {3., 11.4459}}	{47.0005, 59.8165}	inf	18.	68.5032	{8.50129, 56.6683}	304.428	0.386513	newly	6.	56.55	{6., 48.}	852.833	0.631873	Reff	0.548239	0.441198	{0.13285, 1.00664}	48.2638	ar	747.	1617.65	{1439., 2161.}	116.553
31	R0	{2.30764, 13.435}	{2.78191, 6.50443}	{{2.41439, 3.64314}, {3., 11.2145}}	{20.8764, 52.1536}	inf	14.	43.3015	{7.48742, 42.9904}	227.127	0.377491	newly	4.	35.2	{6., 47.}	788.	0.767192	Reff	0.313839	0.431338	{0.185059, 0.71383}	55.9024	ar	877.	1660.31	{1553., 1713.}	89.317
32	R0	{2.30565, 11.9237}	{2.80938, 8.06971}	{{2.40585, 2.75698}, {3., 17.9104}}	{22.1741, 66.2851}	inf	9.	69.7942	{8.35724, 158.377}	687.283	0.689219	newly	6.	102.5	{4., 277.}	1624.	0.82503	Reff	0.341236	0.600402	{0.211929, 1.22513}	99.3813	ar	853.	1785.38	{1567., 2488.}	109.306
33	R0	{2.24031, 18.582}	{2.81272, 9.39546}	{{2.25387, 7.73982}, {3., 11.4899}}	{25.5505, 49.4378}	inf	83.	54.4248	{7.78989, 64.2756}	82.504	0.547513	newly	29.	37.1	{5., 47.}	100.414	0.364607	Reff	0.396535	0.583887	{0.15455, 1.42247}	83.1199	ar	864.	1635.16	{1460., 2200.}	89.2546
34	R0	{2.33306, 15.9759}	{2.63433, 5.99299}	{{2.00889, 0.465791}, {3., 9.96255}}	{24.3583, 63.6126}	inf	34.	55.3233	{6.10306, 51.5575}	112.065	0.328816	newly	12.	70.12	{5., 108.}	505.833	0.492395	Reff	0.354625	0.463201	{0.0696298, 0.61703}	68.7091	ar	854.	1729.26	{1556., 2194.}	102.489
35	R0	{2.48357, 18.3573}	{2.75157, 8.2228}	{{2.45449, 1.1921}, {3., 17.3168}}	{13.7736, 55.6554}	inf	55.	55.7544	{13.4067, 72.3557}	74.3155	0.338048	newly	20.	51.76	{8., 111.}	189.5	0.378788	Reff	0.442075	0.607097	{0.23085, 1.1088}	61.6625	ar	835.	1746.64	{1567., 2057.}	109.178
36	R0	{2.59548, 19.9046}	{2.19518, 4.48457}	{{0.238966, 0.678558}, {3., 15.6476}}	{36.8228, 77.4697}	inf	36.	36.6028	{7.52042, 44.8732}	80.7303	0.371581	newly	9.	43.68	{4., 51.}	406.667	0.511074	Reff	0.399704	0.28915	{0.0417621, 0.475176}	64.6843	ar	863.	1608.71	{1504., 1698.}	86.409
37	R0	{2.29641, 16.9868}	{2.69243, 3.23802}	{{1.75928, 0.69797}, {3., 6.1668}}	{28.7128, 80.938}	inf	41.	30.8665	{2.04678, 36.905}	85.6363	0.537447	newly	12.	48.61	{4., 59.}	334.417	0.463868	Reff	0.314608	0.48341	{0.0774339, 0.939578}	91.629	ar	877.	1491.12	{1373., 1537.}	70.0251
38	R0	{2.65743, 15.4569}	{2.27224, 8.27028}	{{0.116569, 1.42622}, {3., 18.1894}}	{33.4375, 51.2113}	inf	23.	50.1416	{10.8811, 49.1359}	133.122	0.248333	newly	9.	31.77	{6., 42.}	267.	0.471197	Reff	0.318892	0.306274	{0.0144746, 0.477258}	63.8367	ar	897.	1640.31	{1523., 1783.}	82.8662
39	R0	{2.67953, 14.5132}	{2.75789, 8.32587}	{{2.38971, 4.18323}, {3., 15.498}}	{12.6236, 47.1233}	inf	24.	47.9753	{7.25892, 69.4588}	140.763	0.293141	newly	6.	40.83	{5., 44.}	592.167	0.548816	Reff	0.310825	0.511541	{0.119263, 0.857453}	82.8053	ar	894.	1707.78	{1543., 1900.}	91.0268
40	R0	{1.94594, 11.928}	{2.59798, 6.0592}	{{1.93772, 1.57258}, {3., 10.8966}}	{34.7342, 49.2019}	inf	36.	46.5827	{8.98336, 57.6903}	67.3402	0.240044	newly	15.	52.03	{7., 54.}	268.2	0.406602	Reff	0.447566	0.496836	{0.0647309, 1.36099}	77.1291	ar	796.	1698.95	{1562., 1953.}	113.436
41	R0	{1.66297, 10.0047}	{2.85503, 5.79999}	{{2.36968, 0.208773}, {3., 14.5923}}	{71.6824, 74.2659}	inf	25.	32.5238	{6.51213, 47.7938}	95.7289	0.328875	newly	8.	54.24	{3., 82.}	600.	0.573342	Reff	0.53215	0.576201	{0.0395776, 1.28418}	68.0848	ar	716.	1604.61	{1454., 1949.}	124.108
42	R0	{2.91568, 16.4453}	{2.39363, 9.97014}	{{1.05901, 4.86875}, {3., 15.5052}}	{21.4911, 39.8675}	inf	25.	65.2895	{11.7971, 111.227}	182.885	0.418048	newly	14.	42.9	{6., 81.}	229.143	0.470904	Reff	0.23617	0.502651	{0.116855, 0.604564}	134.059	ar	919.	1735.37	{1549., 2171.}	88.8324
43	R0	{2.55898, 15.8889}	{2.72723, 8.32333}	{{2.40912, 3.04839}, {3., 16.2253}}	{14.7955, 50.7417}	inf	46.	44.6135	{5.11337, 63.1452}	86.0148	0.426414	newly	20.	46.67	{3., 63.}	190.55	0.42208	Reff	0.30196	0.553085	{0.247003, 0.739616}	92.7863	ar	904.	1655.59	{1502., 2072.}	83.1405
44	R0	{2.28649, 15.9017}	{2.41899, 9.21565}	{{1.27485, 3.38155}, {3., 17.1952}}	{34.2357, 45.1555}	inf	33.	71.9994	{11.926, 84.0917}	145.03	0.316722	newly	11.	50.32	{7., 90.}	372.545	0.505165	Reff	0.324682	0.475401	{0.13039, 0.868712}	76.2283	ar	880.	1724.98	{1550., 2036.}	96.0205
45	R0	{2.65121, 18.6339}	{2.46992, 6.4859}	{{1.06697, 3.38254}, {3., 7.88783}}	{25.0282, 65.193}	inf	63.	70.9302	{6.64243, 192.727}	110.798	0.52998	newly	16.	75.65	{6., 123.}	400.563	0.444814	Reff	0.230655	0.50268	{0.0578213, 0.968711}	145.54	ar	928.	1746.61	{1546., 2281.}	88.2123
46	R0	{2.52291, 18.0251}	{2.33722, 7.95642}	{{1.24081, 4.17263}, {3., 10.8992}}	{29.6237, 56.2405}	inf	48.	46.1765	{8.59472, 63.1035}	79.9988	0.382537	newly	15.	52.56	{6., 53.}	277.6	0.355047	Reff	0.32041	0.51821	{0.133198, 0.892204}	91.6678	ar	894.	1644.93	{1518., 1700.}	83.9966
47	R0	{2.22618, 17.1225}	{2.57155, 6.93285}	{{2.00639, 0.885838}, {3., 16.4053}}	{26.8724, 59.6954}	inf	64.	51.4274	{3.62712, 72.6532}	80.3824	0.51814	newly	23.	70.24	{4., 110.}	256.261	0.453867	Reff	0.454141	0.499201	{0.109067, 0.949848}	68.1901	ar	850.	1712.61	{1532., 2250.}	101.484
48	R0	{2.04405, 16.073}	{2.65671, 3.71072}	{{2.08518, 1.36623}, {3., 8.97125}}	{33.7591, 76.9134}	inf	45.	36.5339	{4.82931, 54.8567}	102.04	0.59945	newly	18.	90.52	{3., 249.}	454.222	0.572276	Reff	0.533497	0.665735	{0.115332, 1.53198}	88.1825	ar	779.	1698.59	{1501., 2104.}	118.047
49	R0	{2.84396, 18.097}	{2.49983, 7.0149}	{{1.25181, 3.36073}, {3., 8.72795}}	{19.3428, 61.2372}	inf	41.	67.6289	{8.87296, 145.42}	121.469	0.369578	newly	16.	57.67	{6., 117.}	284.313	0.436266	Reff	0.170638	0.582255	{0.146495, 0.954383}	249.49	ar	952.	1809.88	{1600., 2313.}	90.1134
50	R0	{1.85341, 18.0975}	{2.71066, 3.88919}	{{2.18817, 2.00218}, {3., 3.86866}}	{46.2526, 78.5098}	inf	40.	61.3925	{9.38395, 64.9798}	108.261	0.307101	newly	17.	110.23	{8., 293.}	566.882	0.5302	Reff	0.498567	0.617466	{0.126811, 1.57864}	79.6625	ar	754.	1729.77	{1528., 2112.}	129.412
